# Foreword: Focus on memorial issue dedicated to Dr. Allan S. Hoffman: the father of biomaterials

**DOI:** 10.1080/14686996.2025.2504796

**Published:** 2025-05-16

**Authors:** Mitsuhiro Ebara, Takashi Miyata, James Lai

**Affiliations:** a Research Center for Macromolecules and Biomaterials, National Institute for Materials Science, Tsukuba, Japan; b Department of Chemistry and Materials Engineering, Kansai University, Osaka, Japan; c Department of Materials Science and Engineering, National Taiwan University of Science and Technology, Taipei, Taiwan

This special issue aims to honor the extraordinary scientific career of Dr. Allan S. Hoffman (1932–2023), who is also known as the father of biomaterials. These materials were originally designed to support, enhance, or replace damaged tissue or biological functions playing an integral role in medical applications today, and have been widely used throughout medicine, dentistry, and biotechnology. However, the term ‘biomaterial’ was not used in the mid-20th century. Dr. Hoffman pioneered biomedical applications of stimuli-responsive polymers, biocompatible polymers, hydrogels, nanoparticles, bioconjugates, and surface coatings in the fields of drug delivery, separations, biomaterial surface modifications, diagnostic assays, and biologically active and non-fouling implants. He published over 500 scientific papers, including seminal works on temperature-responsive polymers. Dr. Hoffman also led educational campaigns, published textbooks, and devoted tremendous effort to connecting scientists from various fields – including medicine, biology, physics, chemistry, and materials science – thereby fueling the significant growth of the biomaterials field over the past few decades. In recognition of his monumental impact, Dr. Hoffman was named one of the ‘World’s Most Influential Scientific Minds’ by Thomson Reuters in 2015 and was elected to the U.S. National Academy of Engineering in 2005. This special issue features contributions from leading scientists who have either personally known Dr. Hoffman or been inspired by his pioneering work, paying tribute not only to a brilliant researcher but also to a remarkable mentor – widely recognized for training and inspiring many individuals who have gone on to successful careers – and a person who touched the lives of many.

As guest editors, we are confident that this focus issue provides a concise overview of the advancements in the field of biomaterials. Also, we hope this special issue provides a timely opportunity to pay tribute to Dr. Hoffman.

